# Pharmacists’ Interventions on Electronic Prescriptions from Various Specialty Wards in a Malaysian Public Hospital: A Cross-Sectional Study

**DOI:** 10.3390/pharmacy9040161

**Published:** 2021-10-01

**Authors:** Poh Ling Ooi, Hadzliana Zainal, Qi Ying Lean, Long Chiau Ming, Baharudin Ibrahim

**Affiliations:** 1 Department of Pharmacy, Hospital Sultan Abdul Halim, Sungai Petani 08000, Kedah, Malaysia; plooi85@yahoo.com.my; 2 School of Pharmaceutical Sciences, Universiti Sains Malaysia, George Town 11800, Penang, Malaysia; hadz@usm.my; 3 Faculty of Pharmacy, Universiti Teknologi MARA, Cawangan Pulau Pinang, Kampus Bertam, Kepala Batas 13200, Pulau Pinang, Malaysia; leanqiying@uitm.edu.my; 4 PAP Rashidah Sa’adatul Bolkiah Institute of Health Sciences, Universiti Brunei Darussalam, Gadong, Bandar Seri Begawan BE1410, Brunei; longchiauming@gmail.com; 5 Faculty of Pharmacy, University of Malaya, Kuala Lumpur 50603, Selangor, Malaysia

**Keywords:** clinical pharmacy, medication safety, prescribing error, hospital information system, e-prescribing

## Abstract

**Background:** The emergence of new technologies in the area of health information and communication helps pharmacists to check the safety of medications used via electronic prescribing. **Objectives:** The study aimed to identify the rate and types of problems with electronic prescriptions (e-prescriptions) that required pharmacist intervention at an inpatient pharmacy, and to evaluate prescribers’ acceptance of these interventions. **Methods:** A retrospective cross-sectional study on the interventions of e-prescriptions documented by pharmacists was conducted in a public hospital inpatient pharmacy. Data were collected for descriptive analysis using a collection form, including the e-prescription interventions, types of wards, drugs involved, and acceptance of intervention by prescribers. A chi-square test was used to evaluate the association between ward pharmacist availability and the rate of interventions. **Results:** A total number of 11,922 (3.3%) pharmacist interventions were proposed for 357,760 e-prescriptions ordered in the 12 month study period. Of the total number of proposed interventions, 11,381 (95.5%) were accepted by prescribers. The interventions on e-prescriptions were from surgical wards (11.7%) followed by intensive care (5.6%), paediatric (3.5%) and medical specialty wards (2.9%). Anti-infective agents (33.8%) and cardiovascular medicines (27.0%) were among the drugs with the highest rate of interventions. The most common type of intervention was revising the drug regimen (58.4%), especially with anti-infective agents (33.8%). Prescribers in surgical wards showed the highest level of acceptance of pharmacist interventions, which was 97.37%. The presence of ward pharmacists showed a higher number of interventions (6.2 vs. 1.0%, *p* < 0.001) than wards without pharmacists, as well as a higher percentage of acceptance (96.4 vs. 91.1%, *p* < 0.001) towards e-prescription intervention. **Conclusion:** In e-prescribing, errors can be prevented by pharmacists’ interventions on e-prescriptions. This helps to prevent medication errors and thus optimise rational pharmacotherapy in patients. The role of ward pharmacists in pharmaceutical care is highly accepted by prescribers.

## 1. Introduction

In promoting rational evidence-based prescribing, prescriptions will be screened and reviewed by pharmacists before medications are dispensed [1,2]. Studies in different health care settings have found that up to 10% of prescriptions reviewed had problems that needed pharmacists’ intervention [3,4,5,6,7]. Prescription errors requiring pharmacists’ interventions are usually minor errors, though a small number of prescription errors have been reported to cause major harm to patients [8,9]. A pharmacist intervention is defined as any appropriate action taken by a pharmacist to change patient management or therapy [10]. Pharmacist interventions are important in solving medication-related problems that ultimately optimise patients’ pharmacotherapy [10]. Besides preventing medication-related errors and ensuring patients receive the most appropriate medications, interventions by pharmacists can avoid unnecessary costs due to adverse drug events [10].

Similarly, in an inpatient setting, depending on the wards from which the hand-written prescriptions come, such prescriptions have been found to contain problems that need to be rectified or resolved by the ward or inpatient pharmacists [10]. In certain Malaysian hospitals, the prescriptions are usually hand-written and then screened for completeness, prescribers’ authenticity, suitability of drug and dosing regimen, drug interaction, polypharmacy, and the route of administration before dispensing [3,9,10,11,12]. When issues arise, prescribers will be contacted by pharmacists for clarification or amendment of the prescriptions [8]. To smoothen and quicken the process of dispensary at inpatient services, a computerised system that is fool-proof and reliable could serve as an alternative to replace manual order entries made by physicians and to assist in prescription screening, especially when the use of medicines is promptly required for critically ill patients. Any medication errors will potentially cause serious harm, such as a delay in treatment, a complication in a patient’s medical problem(s), a failed therapeutic approach and even death. Therefore, electronic prescribing (e-prescribing) has been promoted as a means to reduce prescribing and medication errors around non-formulary drug entry, absolute drug–drug interactions and patient drug allergies [13].

The escalating use of prescription drugs has increased tremendously over the years with an increasing number of patients with morbidities [14]. The implementation of electronic prescriptions (e-prescriptions) is being applied broadly to increase dispensing efficiency as part of integrated healthcare and medicines safety networks in hospitals or community pharmacies [13,15]. E-prescriptions enable the prescribing, dispensing and claiming of medicines, without the need for a paper prescription. Similarly, the inpatient pharmacy of Sultan Abdul Halim Hospital, one of the public hospitals in Northern Malaysia, has incorporated the Electronic Hospital Information System (e-HIS) as an e-prescription system to improve and thus reduce the risk of medication errors. Nonetheless, there is a lack of studies reviewing the implementation of an e-prescribing system within inpatient pharmacies in local settings. Therefore, this study aimed to evaluate the e-prescription system by identifying the rate and types of problems associated with e-prescription at an inpatient pharmacy that required pharmacists’ intervention and to evaluate the acceptance by the prescribers towards these interventions.

## 2. Methods

### 2.1. Study Design

This was a one-year retrospective, single-centre, cross-sectional study. All interventions on e-prescriptions by pharmacists documented in the inpatient pharmacy (ward supply pharmacy, discharge pharmacy and ward pharmacy) of Sultan Abdul Halim Hospital were recorded. Non-clinical interventions such as an inquiry on legibility and incomplete prescriptions were excluded from this study. Data confidentiality was maintained, including the name of the pharmacist who intervened, the prescriber, and the patient’s identity. The study protocol was submitted to the National Medical Research Register and approved by the Medical Research Ethics Committee, Ministry of Health Malaysia (Code (5) KKM/NIHSEC/P14-1205).

### 2.2. Classification of Data

The e-prescriptions received at the inpatient pharmacy were classified based on the ward specialties: intensive care, medical, paediatric, surgical, orthopaedic, obstetrics and gynaecology, psychiatric and multiple specialties. The e-prescriptions were also divided into two categories, based on the presence or absence of ward pharmacists. Types of e-prescription interventions were categorised into inappropriate or incorrect choice of drug, dose, frequency, duration, polypharmacy, contraindication, and drug interaction. The drugs included in the interventions were pharmacologically categorised according to the British National Formulary.

### 2.3. Collection and Analysis of Data

A structured data collection form was used to collect the data of e-prescription interventions. Data were then entered into Microsoft Excel 2007 and SPSS version 17 (IBM, Armonk, NY, USA) software for further data analysis. Descriptive statistical analysis was used to assess the rate of interventions, types of e-prescription interventions, categories of drugs involved, and acceptance among prescribers. A chi-square test was used to evaluate the association between the presence of a ward pharmacist and the rate of interventions as well as the acceptance among prescribers.

## 3. Result

### 3.1. Rate and Acceptance of Interventions on E-Prescriptions

A total of 357,760 e-prescriptions (Table 1) were received by the inpatient pharmacy from different specialty wards during the one-year study period. Of the total e-prescriptions, 11,922 (3.3%) of reviewed e-prescriptions were proposed for interventions, with 82.7% by ward pharmacists and the rest by pharmacists stationed at the inpatient pharmacy or satellite pharmacies. A significantly higher number (6.2%) of e-prescription interventions were made in the presence of ward pharmacists versus wards without pharmacists (1.0% of total e-prescriptions). The prescribers accepted 11,381 interventions, which was 95.5% of the total e-prescription interventions. In other words, for all e-prescriptions, there was a total of 3.2% interventions in the current study. Separately, there was a significantly higher acceptance rate (96.4%) among prescribers in the wards with the presence of ward pharmacists compared to the rate (91.1%) in wards without ward pharmacists.

Table 2 shows the number of e-prescriptions, interventions, and acceptance in different specialty wards. The greatest number of interventions was on e-prescriptions from surgical wards (11.7%), followed by intensive care wards (5.6%), paediatric wards (3.5%) and medical wards (2.9%). Prescribers in the surgical wards showed the greatest acceptance of pharmacist interventions which was 97.4%, followed by the medical wards (96.6%), orthopaedic wards (92.2%), paediatric wards (91.3%), and wards with multiple specialties (90.9%). The lowest acceptance was from prescribers in the psychiatric wards (60%). 

### 3.2. Types of Interventions on E-Prescriptions

Table 3 provides the common reasons for interventions on the e-prescriptions by pharmacists. The most common type of interventions performed were on drug prescribed (58.35%), followed by dose (18.65%) and frequency (13.73%). Drug interaction (0.03%) and contraindication (0.59%) were the least common issues related to the e-prescriptions.

When comparing the types of interventions at different specialty wards, drug interventions were made for about two thirds of the surgical e-prescriptions while the majority of the specialty wards showed the same trend on the types of interventions carried out by pharmacists (Table 3). The most frequent interventions were on drug choice, followed by dose, frequency, duration, polypharmacy, contraindication, and drug interaction. In contrast, dose was the most common interventions in paediatric wards while frequency was the most common intervention in the orthopaedic wards (Table 3). Only surgical ward reported intervention for drug interaction (0.06%).

### 3.3. Categories of Drugs on E-Prescriptions with Pharmacist Intervention

Overall, anti-infective agents and cardiovascular medications were among the drugs with the highest rate of interventions, respectively 33.84 and 27.00%. Other interventions involved nutritional and blood preparations (8.81%), endocrine agents (8.79%) and gastrointestinal agents (7.39%), and other symptomatic relief medication (14.17%). Table 4 depicts the categories of intervened drugs on e-prescriptions from different specialty wards. Anti-infective agents were the most frequently intervened items on the e-prescriptions for the intensive care (53.76%), orthopaedic (50.94%), paediatric (49.18%), and surgical (41.91%) wards. The most commonly intervened drugs on the medical wards and psychiatric wards were, respectively, cardiovascular medications (36.85%) and central nervous system agents (70.00%). Drugs for musculoskeletal and joint diseases only accounted for 6.22% of the total interventions in orthopaedic wards. Likewise, only 1.26% interventions were made on drugs for obstetrics, gynaecology and urinary tract disorders in obstetrics and gynaecology (O&G) wards.

## 4. Discussion

The current study detected the incidence of inpatient prescribing errors that required active interventions by pharmacists. The rate of e-prescription intervention by pharmacists was 3.2%, which is similar to the rate of interventions (3.8%) reported at chain community pharmacies which had implemented e-prescribing [16] but lower than the intervention rate (7.1%) reported for manual medication charts and orders at an inpatient pharmacy in a teaching hospital within a month [3]. E-prescribing has been advocated as a potential medication error reduction mechanism by studies showing lower rates of interventions [4,17]: 2.3 and 0.68%, respectively, for discharged patients. However, other studies [8] recorded a higher rate of interventions: 9.1 and 10.1%, respectively, at an inpatient pharmacy and at an emergency department for discharge prescriptions with the implementation of computerised prescribing order entry. Up to 45.1% of pharmaceutical interventions were performed on e-prescriptions in Norwegian community and hospital pharmacies [18]. The diverse rates in different studies might be due to the difference in the application of classifications of medication errors in intervention and the adoption of specific medication-prescribing policies in various settings. For example, some interventions are made due to a policy requirement or a technical issue such as a shortage of medication or change to a new generic alternative brand with a lower cost. Nonetheless, overall findings suggest that, with e-prescribing, medication-related problems of clinical importance remain. Thus, screening of prescription and interventions by pharmacists continue to be relevant and crucial.

Generally, any changes made to prescriptions must obtain the consent of the prescriber and be clearly documented. Likewise, in e-prescribing, pharmacists communicate with prescribers to resolve the e-prescribing issues. Ward pharmacists work with other health professionals as a ward team to ensure each patient receives correct medication and to safeguard against medication errors when patients are admitted to, during hospitalisation, and discharged from hospital [13]. Even during the pandemic era, ward pharmacists have integrated several telehealth initiatives to communicate any pharmaceutical-related problems detected and to perform remote pharmaceutical care for COVID-19 patients [2]. When no ward pharmacists are stationed at the specialty wards, there remain screening and interventions by pharmacists when the e-prescriptions reach in-patient pharmacy. Overall, the acceptance rate of pharmacists’ interventions in this study was high (95.5%), indicating doctors were receptive to the pharmacists’ recommendations. Similar results on high acceptance among prescribers were shown in studies by Al Rahbi et al., Li et al. and Kuo et al., which were 98.2, 97.3 and 89%, respectively [3,4,17]. In this study, the acceptance of interventions among prescribers in most of the specialty wards was more than 85%, except the psychiatric ward which recorded 60% acceptance. However, this disparate value might not represent the actual conditions due to very low relative number of e-prescriptions and thus interventions in the psychiatric ward. 

Interestingly, different specialties might have different rates of interventions. In the current study, a higher rate of interventions on e-prescriptions from the surgical, intensive care, paediatric and medical wards were seen in comparison to interventions in the other wards. This concurs with another study in which a higher prescribing error rate was found from the Department of Medicine and Intensive Care Unit [19]. This might be due to the high number of admissions, high volume of work, availability of numerous drug choices, and the complexity of drug regimen for disease treatment in these settings. Besides, studies have revealed that interruptions, distractions, and cognitive load increase the chances of medication errors [9,19,20]. Another important contributing factor to higher prescribing issues might be due to the use of unfamiliar and new prescribing software by the physicians in their practices following the initiation of e-prescribing. It is expected that the rate of interventions will be lower once the prescribers adapt to and become adept at a new prescribing system. 

On the other hand, the rate of interventions on e-prescriptions from paediatric wards in this study was low (3.2%) compared to other wards whereas a study by Cesarz et al. (2013) showed the rate of interventions on paediatric prescriptions (23.6%) was higher than adult prescriptions (8.5%). Nonetheless, it is important to note that the specialty wards with ward pharmacists had a significantly higher number of interventions, likely owing to these pharmacists’ active participation in clinical rounds, better clinical knowledge, and competence in medication use and recommendations, and thus fostering identification of patient medication needs and related problems [20,21,22]. Similarly, the acceptance rate among prescribers in wards with ward pharmacists on duty was significantly higher than in wards without ward pharmacists. The high acceptance rate implies that prescribers are open to pharmacists’ suggestions regarding drug therapy, and work collaboratively to ensure patient safety and optimal health outcome during initiation of treatment, medication review and reconciliation at all points of care.

Medication errors, including with prescribing and dispensing, can occur during the treatment process. Pharmacists have a role in minimising and preventing such errors. The screening of e-prescriptions based on a patient’s condition could be used as a safety net. In a similar fashion, prescribers should implement more measures to review e-prescription entries before submission to an inpatient pharmacy. The majority of e-prescription interventions in this study were made on drug, dose, and frequency. The same trend was also illustrated in several other studies [4,19,21,23]. The e-prescribing system is believed to provide dose checking but it seems the potential of this function is still lacking or underutilised. In addition, common types of interventions might be affected by the setting; for example, a study at an emergency department demonstrated that the common types of interventions conducted by pharmacists were dosage (35%), dilution (9.77%), administration route (8.48%), infusion time (6.13%) and frequency (5.89%) [17]. Medication errors appeared to be reduced over time with interventions but there remain some common types of medication errors related to dose during medicine reconciliation [24].

In this study, the categories of drugs most often undergoing interventions were anti-infective agents and cardiovascular medications, followed by nutritional and blood preparations, endocrine agents, and gastrointestinal agents. Our results concur with other findings [21,25]. The frequent use of cardiovascular drugs is mostly due to the high prevalence of cardiovascular diseases such as ischemic heart disease, the leading cause of death in Malaysia [26]. On the other hand, patients are usually hospitalised due to acute moderate to severe infections, such as pneumonia, which require the use of antibiotics [27]. Therefore, our findings stress the need for reinforcement of cardiovascular and infectious diseases medicine in clinical practice among healthcare professionals. Cesarz et al. also found that the most common categories of drugs intervened at an emergency department were central nervous system agents (29.4%), anti-infective agents (26.5%), gastrointestinal agents (19.2%), and autonomic drugs (7.4%) [24]. All this points to the need for more vigilant use of various groups of agents based on these specialties to prevent prescribing and medication errors. 

Like other studies, although we have seen that e-prescribing eases the prescribing process and improves safety of medicine use, [21,25] our study has reinforced the fact that e-prescribing does not completely eliminate errors that need pharmacists’ judgment. In other words, it is not a substitute for a pharmacist’s role in evaluating prescriptions for essential interventions of clinical importance. The common encountered problems identified with e-prescriptions can be submitted to the e-prescribing system, e-HIS in this case, to improve its usefulness for decision support and functionality of e-prescribing in preventing common prescribing errors [26,27]. Continuous improvement in the quality of the e-prescribing system and interdisciplinary partnership, especially between prescribers and pharmacists, will improve the rational use of drugs and increase safety and clinical outcomes [25].

### Study Limitations

This study was conducted in a single centre which may limit generalisability, as the results obtained do not necessarily reflect the pattern of prescription-associated problems and thus interventions in other hospitals. This was a retrospective study based on computerised records, thus incomplete information and underreporting might exist due to the hectic working environment in the inpatient pharmacy department. A multidisciplinary panel for the assessment of the significance of interventions made by pharmacists and the types of interventions was not available. Future studies could include these aspects to investigate the effectiveness of e-prescribing operation, so that the system could be introduced to other units or different hospitals or healthcare settings. 

## 5. Conclusions

Although e-prescription has been an alternative to smoothen the process of inpatient medication order and dispensing, the risk of prescription errors remains and requires pharmacists’ intervention, though probably at a lower rate than manual prescribing. Pharmacists’ interventions during prescription screening and ongoing communication with prescribers, particularly around drug regimens, are highly warranted to minimise prescription errors. Future strategies for reducing prescribing problems could target the categories of drugs and specialty wards most frequently associated with e-prescription interventions. The role of pharmacists in each ward for the identification of prescription-associated problems should not be reduced and should be generally appreciated by prescribers in optimising pharmaceutical care.

## Figures and Tables

**Table 1:** The number of proposed interventions, and acceptance based on the availability of ward pharmacists on e-prescriptions.

	Total	Ward Pharmacist Availability	
		Yes	No
No. of proposed interventions	11,922	9857 (82.7)	2065 (17.3)
No. of interventions accepted	11,381	9499 (83.5)	1882 (16.5)
Acceptance rate (%)	95.5	96.4	91.1

**Table 2:** The number of prescriptions, proposed interventions, and acceptance on e-prescriptions in different specialty wards.

Ward Specialty	No. of Prescriptions (%)	No. of Prescriptions with Proposed Interventions (%)	Percentage of Prescriptions with Proposed Interventions (%)	No. of Prescriptions with Accepted Interventions (%)	Percentage of Prescriptions with Accepted Interventions (%)	Acceptance Rate (%)
Medical	96,032 (26.8)	2817 (23.6)	2.9	2722 (22.8)	2.8	96.6
Obstetrics and Gynaecology	83,260 (23.3)	477 (4.0)	0.6	429 (3.6)	0.5	89.9
Surgical	53,301 (14.9)	6235 (52.3)	11.7	6071 (50.9)	11.3	97.4
Orthopaedic	41,434 (11.6)	691 (5.8)	1.7	637 (5.3)	1.5	92.2
Intensive Care	10,020 (2.8)	559 (4.7)	5.6	485 (4.1)	4.8	86.8
Paediatric	10,061 (2.8)	355 (3.0)	3.5	324 (2.7)	3.2	91.3
Psychiatric	1607 (0.4)	10 (0.1)	0.6	6 (0.1)	0.4	60.0
Others	62,045 (17.3)	778 (6.5)	1.3	707 (5.9)	1.1	90.9
Total	357,760	11,922	3.3	11,381	3.2	95.5

**Table 3:** Types of interventions on e-prescriptions in different specialty wards.

Types of Interventions	Number (%)	Medical (%)	O and G (%)	Surgical (%)	Orthopaedic (%)	Intensive Care (%)	Paediatric (%)	Psychiatric (%)	Others (%)
Drug	6927 (58.35)	56.02	54.51	68.61	24.31	50.98	27.04	50.00	36.89
Dose	2218 (18.65)	21.19	17.61	13.70	30.54	12.88	47.04	30.00	29.56
Frequency	1633 (13.73)	16.22	15.72	8.39	37.63	12.88	17.18	20.00	23.52
Duration	665 (5.59)	3.16	7.55	6.08	2.75	16.64	4.51	-	4.24
Polypharmacy	375 (3.15)	3.05	3.56	2.45	4.20	5.90	4.23	-	5.40
Contraindication	70 (0.59)	0.35	1.05	0.71	0.58	0.72	-	-	0.39
Drug interaction	4 (0.03)	-	-	0.06	-	-	-	-	-

**Table 4:** Categories of drugs intervened on e-prescriptions in different specialty wards.

Types of Categories (%)	Medical	O & G	Surgical	Orthopaedic	Intensive Care	Paediatric	Psychiatric	Others
Infectious	24.03	31.66	41.91	50.94	53.76	49.18	-	25.45
Cardiovascular system	36.85	24.11	18.48	15.63	17.20	6.01	10.00	34.45
Central nervous system	3.93	2.94	4.46	5.07	2.15	3.83	70.00	4.11
Nutrition and blood	9.91	10.27	7.10	5.93	6.45	15.85	10.00	8.48
Endocrine	8.81	12.58	7.59	8.97	-	3.28	10.00	9.51
Gastrointestinal system	6.92	7.55	15.51	3.76	13.98	2.19	-	5.53
Respiratory system	7.72	1.68	1.16	2.60	2.15	14.75	-	7.97
Musculoskeletal and joint diseases	0.29	3.98	2.31	6.22	1.08	1.09	-	1.29
Skin	0.58	3.98	-	-	-	1.64	-	1.16
Ear, nose and oropharynx	0.66	-	0.17	-	1.08	1.64	-	0.51
Obstetrics, gynecological and urinary tract disorders	0.15	1.26	0.83	0.29	1.08	-	-	0.51
Anaesthesia	-	-	0.33	0.29	1.08	0.55	-	0.13
Eye	0.07	-	-	-	-	0	-	0.90
Malignant disease	0.07	-	0.17	0.29	-	-	-	-
